# The involvement of astrocytes in early‐life adversity induced programming of the brain

**DOI:** 10.1002/glia.23625

**Published:** 2019-04-30

**Authors:** Maralinde R. Abbink, Anne‐Lieke F. van Deijk, Vivi M. Heine, Mark H. Verheijen, Aniko Korosi

**Affiliations:** ^1^ Center for Neuroscience, Swammerdam Institute for Life Sciences, University of Amsterdam Amsterdam The Netherlands; ^2^ Department of Molecular and Cellular Neurobiology, Center for Neurogenomics and Cognitive Research Amsterdam Neuroscience, Vrije Universiteit Amsterdam The Netherlands; ^3^ Complex Trait Genetics, Center for Neurogenomics and Cognitive Research Amsterdam Neuroscience, Vrije Universiteit Amsterdam The Netherlands

**Keywords:** early inflammation, early malnutrition, early stress, GFAP, maternal separation/deprivation

## Abstract

Early‐life adversity (ELA) in the form of stress, inflammation, or malnutrition, can increase the risk of developing psychopathology or cognitive problems in adulthood. The neurobiological substrates underlying this process remain unclear. While neuronal dysfunction and microglial contribution have been studied in this context, only recently the role of astrocytes in early‐life programming of the brain has been appreciated. Astrocytes serve many basic roles for brain functioning (e.g., synaptogenesis, glutamate recycling), and are unique in their capacity of sensing and integrating environmental signals, as they are the first cells to encounter signals from the blood, including hormonal changes (e.g., glucocorticoids), immune signals, and nutritional information. Integration of these signals is especially important during early development, and therefore we propose that astrocytes contribute to ELA induced changes in the brain by sensing and integrating environmental signals and by modulating neuronal development and function. Studies in rodents have already shown that ELA can impact astrocytes on the short and long term, however, a critical review of these results is currently lacking. Here, we will discuss the developmental trajectory of astrocytes, their ability to integrate stress, immune, and nutritional signals from the early environment, and we will review how different types of early adversity impact astrocytes.

**Abbreviations**
ELA: early‐life adversity
CNS: central nervous system
BBB: blood–brain barrier
GFAP: glial fibrillary acidic protein
GR: glucocorticoid receptor
CORT: corticosterone
MD: maternal deprivation
MS: maternal separation
P: postnatal day
LPS: lipopolysaccharides
HFD: high‐fat diet
MDD: major depressive disorder
SCZ: schizophrenia

## 1 INTRODUCTION

Early life is a critical developmental period in which the brain is shaped for life. Early‐life adversity (ELA), in the form of trauma, severe abuse or neglect, prolonged hospitalization, infection, or malnutrition (de Rooij, Wouters, Yonker, Painter, & Roseboom, 2010; Kessler et al., 2010; Loman & Gunnar, 2010; Mandelli, Petrelli, & Serretti, 2015; Nemeroff, 2016), has been associated with cognitive decline and increased risk of developing psychopathology in adulthood. For example, social deprivation in early institutionalized children results in lower cognitive ability (Chugani et al., 2001; Nelson et al., 2007), childhood trauma caused by neglect or emotional abuse is associated with impaired cognitive functioning and adult depression (Mandelli et al., 2015; Saleh et al., 2017), and prenatal undernutrition leads to impaired cognition in adulthood (de Rooij et al., 2010) and increased vulnerability to develop schizophrenia (Brown & Susser, 2008). During early‐life, the brain needs to integrate and adapt to many different environmental factors for proper development. There is increasing attention for the synergistic and coordinated action of multiple early‐life environmental factors, that is, stress hormones, nutritional input, and immune activation, in programming of the brain (Cirulli, 2017; Hoeijmakers, Korosi, & Lucassen, 2015; K. L. Lindsay, Buss, Wadhwa, & Entringer, 2017; Lucassen et al., 2013; Marques, 2013; Marques, Bjørke‐Monsen, Teixeira, & Silverman, 2015; Yam, Naninck, Schmidt, Lucassen, & Korosi, 2015), but the exact mechanisms underlying this process, and in particular what neurobiological substrates and central nervous system (CNS) cell types are involved, remains to be elucidated.

The hippocampus has been extensively studied in this context, because of its key role in cognitive functioning and its high degree of neuronal and synaptic plasticity (Akhondzadeh, 1999; Lledo, Alonso, & Grubb, 2006; Malenka, 1994). There is now ample evidence that ELA in the form of stress or malnutrition leads to impaired cognitive functioning associated with disrupted hippocampal neurogenesis (Abbink, Naninck, Lucassen, & Korosi, 2017; Lemaire, Koehl, Le Moal, & Abrous, 2000; Loi, Koricka, Lucassen, & Joels, 2014; Matos, Orozco‐Solís, de Souza, Manhães‐de‐Castro, & Bolaños‐Jiménez, 2011; Naninck et al., 2015; Pérez‐García, Guzmán‐Quevedo, Da Silva Aragão, & Bolaños‐Jiménez, 2016) and altered synaptic plasticity (Aisa et al., 2009; Austin, Bronzino, & Morgane, 1986; Danielewicz, Trenk, & Hess, 2017; Derks, Krugers, Hoogenraad, Joels, & Sarabdjitsingh, 2016; for review see Georgieff, Brunette, & Tran, 2015; J. Yang et al., 2007). In addition, the involvement of microglia has received some attention (De Luca et al., 2016; Diz‐Chaves, Pernía, Carrero, & Garcia‐Segura, 2012; Hoeijmakers et al., 2017). Although changes in astrocytes have been described in the context of several ELA‐associated psychopathologies like depression and schizophrenia (see Box 1) (Cobb et al., 2016; Webster et al., 2001), the role of astrocytes in ELA programming received only marginal attention.

BOX 1 **Implication of astrocytes in human diseases associated with ELA**

In humans, ELA has been associated with neurological and mental problems later in life, such as major depressive disorder (MDD) and schizophrenia (SCZ). Interestingly, in line with findings in rodent models, many studies in human MDD and SCZ patients point to aberrant astrocyte functions. In MDD patients, especially in the younger subjects, astrocyte pathology is more prominent than neuronal dysfunction, pointing to a role for astrocytes already at early stages of the disease course (Miguel‐Hidalgo et al., 2000). Postmortem studies in MDD patients showed decreased astrocyte numbers and abnormal astrocyte morphology in the frontolimbic system. Decreased GFAP expression and reduced numbers of GFAP+ and S100β^+^ astrocytes are found in the prefrontal cortex (Nagy et al., 2015), anterior cingulate cortex (Gittins & Harrison, 2011), and the hippocampus (Cobb et al., 2016) of MDD patients, brain regions that are specifically involved in mood disorders (Torres‐Platas, Nagy, Wakid, Turecki, & Mechawar, 2016). In SCZ, less consistent changes in astrocytes have been found, while some studies indicated increased (Feresten, Barakauskas, Ypsilanti, Barr, & Beasley, 2013), others found decreased astrocyte numbers in postmortem tissue of SCZ patients (Steffek, McCullumsmith, Haroutunian, & Meador‐Woodruff, 2008). However, in line with the hypothesis that SCZ is related to NMDA dysfunction, and that astrocytes induce release of gliotransmitters, agents that enhance NMDA function such as increasing levels of receptor co‐agonist d‐serine, showed beneficial effects (Heresco‐Levy et al., 2005). Moreover, mutations in Disc1, which protein stabilizes the d‐serine synthesizing enzyme serine racemase, resulted in SCZ‐like behavior in mice (Ma et al., 2013). Anti‐depressant agents in MDD, resulted in recovered astrocyte phenotypes in both rodent models and human patients, and therefore have been argued that the functional improvement of astrocytes should be strategic in the treatment of MDD (Czéh & Di Benedetto, 2013). As many studies support the hypothesis that changes in astrocyte function could contribute to pathophysiology of mood disorders, better understanding of the underlying pathomechanisms involving astrocytes could potentially provide new targets for therapeutics. Additionally, this knowledge could lead to a better understanding of programming of astrocytes by ELA in the context of disease.

Astrocytes are able to sense and integrate a multitude of signals from the endogenous and exogenous environment and use this information to modulate neuronal function. They are involved in many different processes crucial for brain and cognitive functioning (Santello, Toni, & Volterra, 2019) including glutamate recycling (Sonnewald, Westergaard, & Schousboe, 1997), myelination (Camargo et al., 2017; Kıray, Lindsay, Hosseinzadeh, & Barnett, 2016), energy metabolism (Bélanger, Allaman, & Magistretti, 2011), and synaptic development and functioning (Allen, 2014; Allen & Eroglu, 2017). It has been known that astrocytes promote synaptogenesis during development (Allen, 2013), and emerging evidence shows that astrocytes also control dendritic maturation and synaptic integration of adult newborn neurons in the hippocampus (Kempermann, 2015; Sultan et al., 2015). This suggests a possible role for astrocytes in regulating the ELA‐induced alterations in adult neurogenesis (Abbink et al., 2017; Korosi et al., 2012; Naninck et al., 2015). Next to that, astrocytes are important integrative cells that can process information on spatial and temporal scales distinct from those of neurons. Astrocytes release gliotransmitters and interact with both presynaptic and postsynaptic neurons, together forming the tripartite synapse (Araque et al., 2014; Araque, Parpura, Sanzgiri, & Haydon, 1999). Estimations indicate that one single astrocyte can cover and influence up to 140,000 synapses in the hippocampus (Bushong, Martone, Jones, & Ellisman, 2002), allowing the synchronization of neuronal firing and coordination of synaptic networks (Halassa, Fellin, Takano, Dong, & Haydon, 2007). Astrocytes regulate synapse development (Christopherson et al., 2005; Diniz et al., 2012; Kucukdereli et al., 2011) and modulate synaptic transmission within a seconds to minutes timescale (Araque et al., 2014; Parpura et al., 1994; Pascual et al., 2005; Yang et al., 2003). Furthermore, astrocytes modulate the efficiency of excitatory (glutamatergic) synapses by clearing glutamate from the synaptic cleft via two high‐affinity glutamate transporters: The GLT‐1 and GLAST transporters (EAAT2 and EAAT1, respectively) thereby preventing excitotoxicity (damaging of neurons by excessive stimulation of neurotransmitters; Choi, Maulucci‐Gedde, & Kriegstein, 1987; Huang & Bergles, 2004). Finally, astrocytes are sensitive to changes in the environmental state and can respond by regulating extracellular factors, like metabolite concentration, neurotransmitters, and ions (Bazargani & Attwell, 2016; Vernadakis, 1996; Walz, 1989). Astrocytes receive this homeostatic information from different sources and are capable of responding to more “slow” homeostatic processes, including hormonal changes, immune signals, and nutritional input.

Thus astrocytes are in the unique position to sense, integrate, and coordinate the large variety of signals from the complex early‐life environment. Therefore, we propose that ELA might impact on astrocytes and thereby contribute to lasting effects of ELA on brain development, structure, and function. Here, we will describe the timing and origin of astrocyte development, highlight the sensing and integrative nature of astrocyte functions, and review the existing literature on the effects of ELA, for example, stress, inflammation, and malnutrition, on the astrocyte population.

## 2 ASTROCYTE DEVELOPMENT

Astrocytes play a crucial role in normal brain development (Reemst, Noctor, Lucassen, & Hol, 2016), and knowledge on the timing of astrocyte development and maturation is crucial to comprehend how ELA might influence astrocytes and program their function for life. In rodents, astrocytes are derived from three sources: (a) neural progenitor cells, (b) directly from radial glia (Kriegstein & Alvarez‐Buylla, 2009; Marshall, Suzuki, & Goldman, 2003), or (c) in later stages, via local proliferation of differentiated astrocytes (Ge, Miyawaki, Gage, Jan, & Jan, 2012). Astrocytes begin to develop in the final weeks of embryonic development and increase rapidly in number in the first month of life. In the hippocampus, most astrocytes are being generated during the second postnatal week (Catalani, Sabbatini, Consoli, & Cinque, 2002; Nixdorf‐Bergweiler, Albrecht, & Heinemann, 1994). In the first postnatal week, astrocytes start to grow filopodial processes that form a meshy network with physical overlap of astrocytic processes and no clear‐bordered astrocytic domains (Bushong, Martone, & Ellisman, 2004). At the end of the first week, astrocytes in the hippocampal formation start to ramify and gain increased cytoskeletal complexity, which extends into adulthood (Bushong et al., 2004; Catalani et al., 2002; Setkowicz, Pawliński, & Ziaja, 1999). The distinction of spatial astrocytic domains becomes more clear in the second week of development and is profoundly present at the end of the third week (Bushong et al., 2004). After the peak of astrogenesis in the first two postnatal weeks, proliferative capacity drops radically, though a moderate increase in astrocyte number still occurs until 1 month of age (Catalani et al., 2002; Setkowicz et al., 1999). During week 3 and 4 of postnatal development, astrocytes appear to have a more mature phenotype (Bushong et al., 2004). In humans, the peak of astrogenesis is believed to already start during the last phase of gestation (Menassa & Gomez‐Nicola, 2018; Mottahedin et al., 2017; N. Patro, Naik, & Patro, 2015; Semple, Blomgren, Gimlin, Ferriero, & Noble‐Haeusslein, 2013) and to last throughout the postnatal period, when the number of GFAP^+^ cells continues to increase in every part of the CNS (Roessmann & Gambetti, 1986). Thus, in both human and rodents, astrogenesis coincides with early sensitive periods rendering astrocytes particularly sensitive to ELA. Therefore, it is important to consider the impacts of ELA on astrocytes and how these might contribute to ELA‐induced brain dysfunction.

## 3 ASTROCYTES INTEGRATE SIGNALS FROM THE ENVIRONMENT

As introduced earlier, astrocytes integrate a multitude of signals to adapt to the early environment. These signals are partly local (e.g., neurotransmitters (Araque et al., 2014), and largely systemic (e.g., nutrients, hormonal changes (Garcia‐Segura & McCarthy, 2004) related to metabolism (Chowen et al., 2016; Marina et al., 2017; Welcome, 2018), inflammation (Farina, Aloisi, & Meinl, 2007), and stress (Carter, Hamilton, & Thompson, 2013; Paukert et al., 2014). These signals are fundamental during development and need to be integrated for an informed modulation of neuronal development and function throughout life. We will discuss here how astrocytes are capable of sensing and integrating nutritional, immune‐ and stress‐related signals.

### 3.1 Astrocytes sense nutrients and provide them to neurons

During development, the brain is the fastest developing organ with an incredibly high demand for energy and nutrients. A deficit in nutrient availability during early development has lasting consequences for brain function later in life (Crawford, Hassam, & Stevens, 1981; de Rooij et al., 2010; Roseboom, de Rooij, & Painter, 2006). Astrocytes can sense and take up nutrients from the periphery thanks to their strategic anatomical location. Blood vessels in the CNS are ensheathed by astrocyte endfeet that regulate blood–brain barrier (BBB) permeability and coordinate the entrance of nutrients into the brain (Abbott, Rönnbäck, & Hansson, 2006; Alvarez, Katayama, & Prat, 2013; García‐Cáceres, Fuente‐Martín, Argente, & Chowen, 2012). We will highlight the involvement of perivascular astrocytes in glucose and lipid sensing, the most extensively studied nutrients up to date.

Glucose is one of the most important energy sources of the brain. Astrocytes sense systemic glucose levels and take up glucose from the blood via the glucose transporter 1 (GLUT1), present in the endfeet of perivascular astrocytes. Once glucose has passed the BBB, perivascular astrocytes take up glucose and store it as glycogen (Kacem, Lacombe, Seylaz, & Bonvento, 1998; Marina et al., 2017; Morgello, Uson, Schwartz, & Haber, 1995; Welcome, 2018), which can be directly mobilized as energy source by various neurotransmitters like noradrenaline (Pellerin, 2013; Sorg & Magistretti, 1991). Importantly, astrocytes are the only cells in the brain capable of storing glucose as glycogen. It has been suggested that, under high‐energy demanding conditions, astrocytes can also convert glucose into lactate as an alternative source of energy for neurons (Matsui et al., 2017; Pellerin et al., 1998). However, this concept is subject of debate (Chih & Roberts, 2003; Pellerin & Magistretti, 2003)

Next to being involved in glucose metabolism, astrocytes have been shown to play an active role in brain lipid metabolism. Lipids are crucial for brain development and function, and a lack of lipids during early development has deleterious consequences for later brain structure and function (Crawford et al., 1981). The CNS is highly enriched in lipids (Goyal, Iannotti, & Raichle, 2018; Sastry, 1985) which serve as building blocks of neuronal membranes including neurite membranes, synaptic membranes, and myelin sheaths. Specifically polyunsaturated fatty acids (PUFAs, like DHA and AA) have been shown to be crucial for neurite outgrowth (Calderon & Kim, 2004), synaptic transmission (Connor, Tenorio, Clandinin, & Sauvé, 2012), and neurogenesis (Maekawa et al., 2009). In particular, there is evidence that neurogenesis is modulated by factors related to astrocyte fatty acid metabolism, including fatty acid binding proteins and fatty acid synthase (Boneva et al., 2011; Knobloch et al., 2013; Young, Heinbockel, & Gondré‐Lewis, 2013). Also, cholesterol, a structural component of myelin, is an indispensable building block required for normal brain functioning and synaptogenesis (Orth & Bellosta, 2012), and defective astrocyte cholesterol metabolism can contribute to neurological problems (Camargo et al., 2012; Valenza et al., 2015). The uptake of peripheral lipids by the brain has been subject of debate (Schönfeld & Reiser, 2013). Certain circulating peripheral lipids (e.g., PUFAs) might freely enter the brain (Giles et al., 2016; Nguyen et al., 2014), while others (e.g., cholesterol and phospholipids) are prevented from entering by the BBB and need to be synthesized within the brain (Edmond, 2001; Jurevics & Morell, 1995). In contrast to neurons which are inefficient in lipid synthesis, astrocytes are highly active in de novo lipid synthesis (Camargo et al., 2012; Hofmann et al., 2017; Nieweg, Schaller, & Pfrieger, 2009; Pfrieger & Ungerer, 2011; van Deijk et al., 2017) and subsequent transport of lipids to neurons (Boyles, Pitas, Wilson, Mahley, & Taylor, 1985; Mauch et al., 2001; Nieweg et al., 2009; Pfrieger & Ungerer, 2011). For example, astrocytes produce cholesterol (Camargo et al., 2012; Goritz, Mauch, & Pfrieger, 2005; Mauch et al., 2001; van Deijk et al., 2017) and actively elongate and desaturate fatty acid precursors in order to synthesize and release PUFAs (Green & Yavin, 1993; Moore, 2001; Moore, Yoder, Murphy, Dutton, & Spector, 1991). Furthermore, astrocyte lipid uptake via lipoprotein lipase is crucial for body weight control and the regulation of energy homeostasis (Gao et al., 2017).

Considering the high nutrient and energy demand for brain development, impacts of ELA on astrocyte capacity to provide building blocks to neurons during this critical period could contribute to the lasting effects of ELA on brain structure and function. Currently, we lack the knowledge of whether these functions of astrocytes are affected by ELA, and this calls for further investigation on these aspects.

### 3.2 Astrocytes sense and generate inflammatory signals

ELA leads to alterations in the neuroimmune profile and early‐life infection is an important form of ELA that causes lasting alterations in the brain (Bilbo, 2009; Bilbo & Schwarz, 2012; Ganguly & Brenhouse, 2015). Astrocytes act synergistically with microglia in regulating brain immunity and thereby modulate neuronal and synaptic function. Like microglia, astrocytes are capable of producing cytokines and chemokines, and might even act as antigen presenting cells (reviewed in Y. Dong & Benveniste, 2001). Microglia are thought to be the initial responders to immune signals in the brain and release an array of cytokines upon activation (reviewed in [Hanisch, 2002]). Accordingly, the microglia‐induced cascade can modulate astrocytic cell properties by inhibiting gap junctions (Même et al., 2006), stimulating proliferation (Giulian, Li, Leara, & Keenen, 1994), and inducing a reactive state (Balasingam, Dickson, Brade, & Yong, 1996). This reactive phenotype of astrocytes consists of cellular hypertrophy via intracellular glial fibrillary acidic protein (GFAP) upregulation (Hol & Pekny, 2015; Pekny & Pekna, 2014). This process known as reactive gliosis or astrogliosis can, in severe situations, lead to increased proliferation and glial scar formation, that is, creation of a boundary between the affected and the healthy tissue (Sofroniew, 2009). Furthermore, under pathological conditions, reactive astrocytes can start to produce glutamate and GABA which can cause perturbations in synaptic plasticity, with possible consequences for cognitive functioning (S. Jo et al., 2014). Recent evidence classifies two types of reactive astrocytes: A neurotoxic subtype (A1) triggered by the microglia released cytokines interleukin 1α (IL‐1α), TNF, and complement component 1q (C1q), and a protective subtype (A2). The protective A2 astrocytes upregulate neurotrophic factors and promote neuronal survival, while the destructive A1 astrocytes upregulate classical complement cascade genes destructive to synapses and release a neurotoxin that rapidly kills neurons and oligodendrocytes (Liddelow et al., 2017; Zamanian et al., 2012). Importantly, next to instructive functions of microglia towards astrocytes, astrocytes can direct microglial functioning as well. Astrocyte‐derived factors regulate microglial activation (M. Jo et al., 2017; Norden, Fenn, Dugan, & Godbout, 2014; Rocha, Cristovão, Campos, Fonseca, & Baltazar, 2012), phagocytosis (Jeon et al., 2012), and promote microglial synapse engulfment (Vainchtein et al., 2018). Astrocytes also actively phagocytose synapses themselves (Chung et al., 2013). Thus, microglia and astrocytes act synergistically in regulating brain immunity. There is initial evidence that astrocyte function is altered after exposure to activated microglia, indicating that an early‐life inflammatory event might program astrocyte function (Henn, Kirner, & Leist, 2011). However, how ELA affects astrocyte–microglia communication and brain immunity is still in its infancy.

### 3.3 Astrocytes sense stress‐related signals

Stress‐hormone modulation is a central aspect to almost any form of ELA. As stress hormones enter the brain via the BBB, astrocytes are the first brain cells to encounter glucocorticoids (Pretorius & Marx, 2004) and bind to them via the glucocorticoid receptor (GR; Bohn, Howard, Vielkind, & Krozowski, 1991). Multiple effects of glucocorticoids on astrocytes have been described, including alterations in morphology and glucose and glutamate metabolism. Administration of stress‐hormone corticosterone (CORT) to adult rodents has been shown to lead to a global decrease in GFAP expression (O'Callaghan, Brinton, & McEwen, 1989; Zhao & Wang, 2015) while lowering of CORT via adrenalectomy resulted in increased GFAP levels (O'Callaghan et al., 1989). Furthermore, in vitro studies demonstrated that CORT inhibits astrocyte proliferation (Crossin, Tai, Krushel, Mauro, & Edelman, 1997). At the metabolic level, glucocorticoids inhibit glucose transport and decrease glycogen content in cultured astrocytes (Tombaugh, Yang, Swanson, & Sapolsky, 1992; Virgin et al., 1991) indicating alterations in energy metabolism that may result in problems of energy supply to neurons. Under stressful conditions, glutamate levels are elevated, which in turn could affect synaptic transmission (Lowy, Wittenberg, & Yamamoto, 1995; Musazzi et al., 2010; Popoli, Yan, McEwen, & Sanacora, 2011; Raudensky & Yamamoto, 2007; Reagan et al., 2004; Venero & Borrell, 1999). This data shows that stress hormones can affect many crucial functions of astrocytes required for normal brain functioning. Although several effects of glucocorticoids on astrocytes have been described during adulthood, the ELA‐induced impact on astrocytes and the role of glucocorticoids in this still need consideration.

## 4 ELA INDUCED ALTERATIONS IN ASTROCYTES

In the context of ELA, which is often a synergistic action of several environmental signals, it is important to consider that the above‐discussed nutrient, immune, and stress‐related signals are not acting solo, but that their effect on astrocytes is most likely determined by their interaction. Astrocytes are in the unique strategic position to integrate this multitude of signals from the complex early‐life (micro‐)environment, which might be crucial in programming of the brain by early‐life stress and nutrition. Therefore, it is of importance to unravel if and how the early‐life environment shapes astrocytes and whether this contributes to the ELA‐induced risk to develop brain disorders.

Because studying the underlying mechanisms of ELA‐induced effects on the brain remains challenging in humans (see Box 2), rodent models of early‐life stress have been instrumental. ELA models include maternal deprivation (MD) or maternal separation (MS) models, malnutrition in the form of an unhealthy diet or nutrient restriction, and early systemic immune challenges. Finally, other models of ELA have occasionally been used to study early‐life stress (e.g., dexamethasone, noise). Here we will review and discuss how different ELA models affect astrocytes. Most studies on the role of astrocytes in ELA have used GFAP expression as a marker for astrocytes. For the interpretation of these studies, it is important to note that GFAP does not label all astrocytes present in the brain and that changes in the number of GFAP^+^‐astrocytes might not reflect an actual change in general astrocyte number, but rather astrocytes altering their expression profile and thereby downregulating or upregulating their GFAP expression (Tynan et al., 2013).

BOX 2 **Human‐based iPSC models to study astrocytes in the context of ELA**

ELA increases the risk of later life psychopathology, but also genetic vulnerability for neuropsychiatric disorders should be taken into account. Neuropsychiatric disorders are associated with a polygenic risk, which challenges the development of representative models in rodents, and indicates the need for human‐based model systems. Genome‐wide association studies (GWAS) for SCZ and MDD have indicated a role for hundreds of genes in the disorders (D. M. Howard et al., 2018; Schizophrenia Working Group of the Psychiatric Genomics Consortium, 2014). In recent years, the discovery of induced pluripotent stem cell (iPSC) technology has provided new opportunities to generate genetically complex and human‐specific models. iPSC models contain the entire genetic background of the patient donor cells, thereby allowing co‐interactive studies between genetic and environmental changes. This provides relevant options to study how interaction of the genetic background and environmental adversity could lead to pathology.

Many studies in recent years proved the power of iPSC technology. IPSC‐based models identified a number of cell‐autonomous deficits underlying SCZ including astrocyte involvement (Gonzalez, Gregory, & Brennand, 2017; Windrem et al., 2017). Taking into account recent GWAS studies for MDD, iPSC‐based studies for MDD will soon follow. However, considering recent insights in increased astrocyte heterogeneity, including expression profile, structure, and function (Schitine, Nogaroli, Costa, & Hedin‐Pereira, 2015), we need more insight into astrocyte subtypes involved in disease, to recapitulate findings in vivo. In summary, astrocyte dysfunction is apparent in neuropsychiatric diseases such as MDD and SCZ and these disorders have been associated with ELA. Increased knowledge of basic astrocyte biology and their response to environmental factors could contribute to understanding early‐life programming of astrocytes in the context of disease. iPSC models could provide an opportunity to gain more knowledge on basal astrocyte functioning and their response to environmental factors, while also taking into account the genetic information. Eventually, this could lead to a better understanding of how astrocytes might be programmed by ELA.

### 4.1 Maternal deprivation or separation effects on astrocytes

MD and MS are among the most frequently used models to study early‐life stress‐induced alterations. The models discussed here consist of either MD: A single deprivation episode of 4 or 24 hr, or MS: Daily 3‐ or 4‐hr separation for 1–2weeks.

In male rats, MS and MD resulted in decreased GFAP reactivity and reduced cytoskeletal complexity in the hippocampus (Roque, Ochoa‐Zarzosa, & Torner, 2016; Saavedra, Fenton Navarro, & Torner, 2018) and cortex (Musholt et al., 2009) within 24 hr after MS or MD. Similarly, in *Octodon degus*, MD resulted in decreased density of GFAP^+^ astrocytes in the cortex, associated with reduced length and ramification of astrocytic processes (Braun, Antemano, Helmeke, Büchner, & Poeggel, 2009). Interestingly, this was accompanied by an increased number of S100β^+^ cells (Braun et al., 2009), indicating a differential effect of stress on the expression of different astrocyte markers. In contrast to the reduced GFAP signal measured within 24 hr after the stressor, when GFAP expression was measured a few days or weeks after the end of the deprivation or separation period, an increase in the number of GFAP^+^ cells or GFAP expression was found in the hippocampus (Llorente et al., 2009; Réus et al., 2018), cerebellum (Llorente et al., 2009), and prefrontal cortex (Kwak et al., 2009). Together these results show that MD/MS leads to an acute reduction in GFAP, followed by an increase of GFAP when a longer period between stress and measurement was applied. This shift might be explained by the dynamics of the ELA‐induced CORT response and the fact that MD/MS takes place during the peak period of astrogenesis. As mentioned earlier, CORT has been shown to reduce levels of GFAP acutely in adult rodents (Crossin et al., 1997; O'Callaghan et al., 1989; Zhao & Wang, 2015), thus the MD/MS‐induced rise in CORT might lead to the observed acute decrease in GFAP. When the early‐life stress‐induced rise in CORT is normalized, GFAP expression might no longer be suppressed, resulting in the observed catch‐up in GFAP expression, which could be a compensatory response or even a developmental shift in astrogenesis.

When studying the long‐term effects of MD/MS on astrocytes, no changes in astrocyte density, morphology, or GFAP mRNA were reported up to 2 months following ELA (Burke et al., 2013; Gosselin et al., 2010; Lewis, Darius, Wang, & Allard, 2016), with only one study reporting a slight reduction in GFAP^+^ astrocyte density in 12‐month‐old MD‐exposed rats (Leventopoulos et al., 2007). This supports the idea that GFAP levels return back to baseline after an initial response to ELA, with a possible interaction of ELA and aging, which remains to be investigated.

Next to measuring GFAP, a few studies have looked at astrocyte‐specific glutamate transporters and describe that daily MS permanently increases GLAST and GLT‐1 expression in hippocampal astrocytes of 10‐week and 18‐month‐old rats (Martisova et al., 2012). However, a different ELA model in the form of limited nesting and bedding material has been shown to actually decrease GLAST expression and impair astrocytic glutamate uptake in the hypothalamus, which was associated with dysfunctional glutamatergic transmission (Gunn et al., 2013). This suggests that in contrast to GFAP expression, astrocyte‐specific glutamate transporters do seem to be persistently altered, although the direction of the effect might be brain‐region and ELA‐model dependent. As to the possible mechanisms leading to the altered transporter expression, it is interesting to consider the role of glucocorticoids. Both acute and chronic stress in adulthood lead to increases in glutamate (Mayhew, Beart, & Walker, 2015; Popoli et al., 2011) and in vivo and in vitro work have demonstrated that glucocorticoid exposure leads to acute elevations of GLT‐1 expression and increased glutamate uptake by astrocytes. We can speculate that the observed MS/MD‐induced upregulation of astrocytic glutamate transporters may be a response to clear possible excessive glutamate. Indeed, this is supported by (Autry et al., 2006; Reagan et al., 2004; Zschocke et al., 2005), but see (Virgin et al., 1991). An alternative or parallel mechanism involved could be a more direct influence of GFAP levels. In fact loss of GFAP has been linked to increased protein levels of GLT‐1 in the hippocampus, but an inability of the astrocyte to traffic the GLT‐1 transporter to the cells surface, leading to impaired glutamate uptake (Hughes, Maguire, McMinn, Scholz, & Sutherland, 2004). Arguably, the ELA‐induced CORT and early loss of GFAP could lead to altered glutamate transporter expression and function, resulting in persistent alterations in glutamate metabolism possibly in a brain region‐dependent fashion.

**Figure 1:**
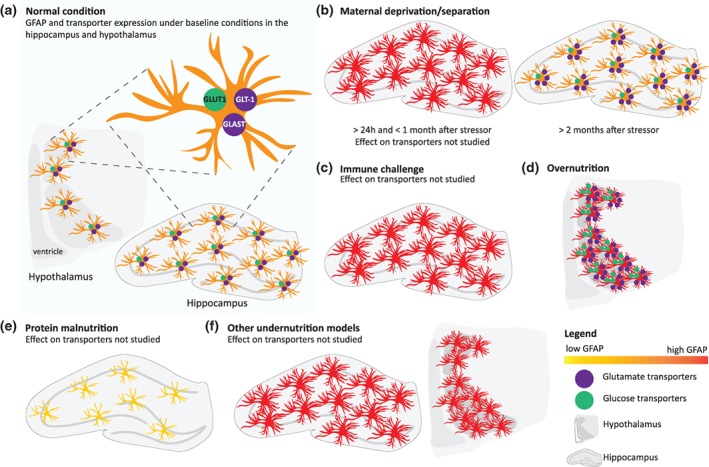
The long‐term effects of ELA on astrocyte characteristics. This figure summarizes the lasting effects of different forms of ELA on the expression of GFAP+ astrocytes and glutamate and glucose transporters. In panel a, a healthy astrocyte under basal conditions is depicted. The remaining panels represent deviations from this following the various forms of ELA. For the conditions where transporter expression was not studied, transporter expression is not included in the figure. (a) Astrocyte under healthy basal conditions in hippocampus and hypothalamus express GFAP, glutamate transporters (GLT‐1 and GLAST), and glucose transporters (GLUT1). (b) MD and MS increase GFAP on the short‐term. On the long‐term, while GFAP returns to basal levels, there is a persistent increase in glutamate transporters. (c) Early‐immune challenge increases GFAP expression. (d) Overnutrition increases GFAP, glutamate transporter, and glucose transporter expression. (e) PMN decreases GFAP expression. (f) Other undernutrition models increase GFAP expression

Although the results derive from a relatively limited number of studies, taken together, early‐life stress in the form of MD or MS seems to affect GFAP expression on the short‐term, and lastingly alter the expression of astrocytic glutamate transporters (see Figure 1b).

### 4.2 Early‐life immune challenge effects on astrocytes

Immune challenges can form a major stressor in early life and considering astrocytic involvement in the immune response as discussed earlier, programming or priming of the astrocyte response to later immune encounters can have implications for brain health in adulthood. One form of an immune stressor is postnatal infection. Rodent studies of neonatal infections using lipopolysaccharides (LPS), polyinosinic:polycytidylic acid (Poly I:C), *Escherichia coli* (*E. coli*), or IL‐6 exposure, reveal astrocytic responses to an early‐life inflammatory challenge. Both LPS and Poly I:C injected at P3, evoked GFAP^+^ cell hypertrophy and increased GFAP^+^ astrocyte numbers in the hippocampus until the age of weaning (N. Patro, Singh, & Patro, 2013), and lasting upregulation of GFAP by LPS was reported at 3 months of age (Berkiks et al., 2019). These effects were accompanied by microglial activation. Postnatal *E. coli* exposure at P4 did not influence astrocytic proliferation in either the hippocampus or cortical subregions at P33, either suggesting that early‐immune challenges, in general, do not affect astrocyte proliferation, that the effect is immune challenge‐specific, or that earlier effects were normalized by P33. Indeed, microglial activation was present at P4 immediately following *E. coli* exposure, an effect that was normalized by P33 (Bland et al., 2010). Prenatal infection induced by IL‐6 exposure resulted in increased astrocyte density and GFAP mRNA levels in the hippocampus at 24 weeks of age (Samuelsson, 2005).

Thus, early‐life immune challenges generally result in lastingly increased levels of GFAP (see Figure 1c) accompanied by microglial activation. As LPS‐induced neuroinflammation is not only a model for early‐life stress but also a well‐described model for microglia‐induced astrogliosis (Liddelow et al., 2017; Zamanian et al., 2012), it could be argued that the observed effects of early immune challenges on astrocytes work through microglia‐induced activation of astrocytes. Early infection could activate microglia, which in turn recruit astrocytes, leading to a reactive phenotype of astrocytes. At this stage, more research is necessary to elucidate the specific order of events. Interestingly, also MD/MS induced alterations on astrocytes were sometimes accompanied by activation of microglia (Réus et al., 2018; Roque et al., 2016; Saavedra et al., 2018). It is striking that despite the similar activation of microglia, different effects were described for GFAP, with lasting upregulation after immune challenges but no persistent changes in GFAP after MD/MS, suggesting alternative mediators and processes involved. This stresses the complexity of the integration of external signals early in life and indicates the importance of the ELA model chosen to study astrocytes.

It is possible that alterations in astrocytes caused by ELA, either through MD/MS or an inflammatory result, are not always detectable under baseline conditions and only become apparent when exposure to another stressful event occurs, a so‐called “second hit.” Evidence for this is provided by studies showing that MS or prenatal restraint stress‐exposed offspring exhibit greater vulnerability of astrocytes to an immune challenge later in life (Diz‐Chaves et al., 2012; Saavedra et al., 2018). This data suggests that astrocytes are sensitized or “primed” by ELA, possibly underlying greater susceptibility to later disease states. ELA might indeed induce a general proinflammatory state of glial cells that may result in an exaggerated reaction to later life infection. Such a stress‐induced sensitized state to later inflammatory challenge has been reported for microglia (Frank, Baratta, Sprunger, Watkins, & Maier, 2007; Frank, Thompson, Watkins, & Maier, 2012; Hoeijmakers et al., 2017). Whether priming of astrocytes is adaptive or maladaptive remains up for debate (García‐Cáceres et al., 2010). In any case, altered sensitization of glial cells by ELA might be a key mechanism in the ELA‐induced increase in vulnerability to cognitive decline and psychopathology development. Finally, it needs to be considered that effects of ELA, next to stress and inflammatory signals, may also be modulated by other factors from the early‐life environment, including dietary and metabolic changes. These factors might interact with one another and contribute to the resulting phenotype.

### 4.3 Early‐life nutrition induced programming of astrocytes

#### 4.3.1 The effects of high fat/sugar diet on astrocytes

In recent years, the effects of an unhealthy, over nutritious high‐fat and/or high‐sugar diet on brain functioning, and the involvement of astrocytes has received considerable attention. These studies mostly focused on hypothalamic astrocytes, as they are key in detecting alterations in metabolic parameters and hormonal changes (e.g., leptin and ghrelin) related to the energy status of the brain (Buckman & Ellacott, 2014; García‐Cáceres et al., 2016; Marina et al., 2017), and have been shown to control food intake (Buckman et al., 2015; Parsons & Hirasawa, 2010; Yang, Qi, & Yang, 2015). To study the effects of an early unhealthy diet, a model of maternal high‐fat diet (HFD) is commonly used. HFD is provided to rodent dams 6–8 weeks before onset of pregnancy and lasts throughout gestation and often also throughout lactation, meaning that nutrients are transferred to the pups via the placenta and breast milk. Maternal HFD leads to an immediate increase in GFAP^+^ cell density, proliferation, and mRNA levels in the arcuate and supraoptic hypothalamic nuclei of mouse neonates (gestational day 17.5 and P0; D. W. Kim, Glendining, Grattan, & Jasoni, 2016), and results in increased levels of perivascular GFAP coverage in the arcuate nucleus of P21 rats, suggesting elevated density of astrocytic processes around the blood vessels (Couvreur et al., 2011). Persistent effects of an early unhealthy diet on astrocytes have also been reported. Postnatal overnutrition in rats as induced by small litter size resulted in increased body weight associated with increased GFAP^+^ cell complexity and cell number, and upregulated protein levels of GFAP (Argente‐Arizón et al., 2017; Fuente‐Martín et al., 2013), VIMENTIN, GLAST, GLT‐1, and glucose transporters GLUT‐1 and GLUT‐2, in the hypothalamus of 3‐ to 5‐month‐old male rats (Fuente‐Martín et al., 2013). Furthermore, a maternal HFD unbalanced in ω‐6/ω‐3 ratio given throughout gestation and lactation, resulted in long lasting increased complexity of GFAP^+^ cells in the hippocampus of 5‐month‐old male rats (Lépinay et al., 2015), however, it is unclear whether these changes arise from a lack of ω‐3 or in response to high‐fat intake.

In general, early‐life HFD or overnutrition seems to acutely and lastingly enhance GFAP expression, and persistently increase astrocyte‐specific glutamate and glucose transporters in the hypothalamus (see Figure 1d). These data suggest possible functional changes in astrocytes with respect to glutamate clearance and nutrient sensing. Interestingly, both early‐life HFD and early‐immune challenges result in increased GFAP expression. Indeed, HFD has been clearly marked in the literature to lead to a general inflammatory response (Buckman et al., 2014) with increased microglial activation (Kälin et al., 2015) and astrogliosis (Balland & Cowley, 2017; Cano et al., 2014; Dalvi et al., 2016) in the hypothalamus. While microglia have been proposed as the driving force behind HFD‐induced obesity (André et al., 2017; Valdearcos et al., 2017, 2014), there was no change or even a decrease in the microglia coverage in the early overnutrition models that we discussed (Argente‐Arizón et al., 2017; Fuente‐Martín et al., 2013), suggesting that the observed changes in astrocyte markers may arise independently from diet‐induced effects on microglia. Moreover, astrocytic changes upon HFD might actually be beneficial for maintaining metabolic homeostasis. Deletion of astrocyte‐specific leptin receptors increased feeding in response to fasting or ghrelin administration, and reduced food intake suppression in response to leptin administration (Kim et al., 2014), indicating the crucial role of leptin receptor signaling in astrocytes for energy homeostasis. Next to that, leptin promotes hypothalamic astrogenesis during development (Rottkamp et al., 2015), which might explain the upregulation of astrocyte markers in response to maternal HFD.

#### 4.3.2 Undernutrition

Models of undernutrition can vary from general food restriction to eliminating specific essential nutrients from the diet. Here we will discuss the effects of early‐life: (a) protein malnourishment (PMN), (b) restriction of essential nutrients, (c) general food restriction as induced by limiting amounts of chow, and (d) early‐weaning (a reduction in maternal milk yield triggered by prolactin injections), on astrocytes. In the PMN models discussed here, protein‐restricted animals received 5–8% of protein versus 20–25% protein in the control groups. Both short‐term (first 2 weeks of gestation) and long‐term (before pregnancy until P60) PMN leads to a decrease in GFAP^+^ cell density, delayed astrogenesis, and precocious maturation of astrocytes in the hippocampus of P60 offspring (see Figure 1e; Gressens et al., 1997; Naik, Patro, Seth, & Patro, 2017), while no significant change in GFAP or S100β seems to be present at the protein level (Feoli et al., 2008). Interestingly, PMN has been shown to result in some acute metabolic alterations, including decreased glutamate uptake, increased glutamate synthetase (GS) activity, and reduced glutathione (a major antioxidant compound metabolized by astrocytes) in the hippocampus and cortex (Feoli et al., 2006). However, these effects present in P2 offspring were normalized by the age of P60. Together these results suggest that PMN influences astrocyte metabolism acutely and induces delayed astrogenesis.

Another model of early‐life malnutrition entails restriction of essential nutrients (nutrients that need to be obtained by the diet). Restriction of ω‐3 PUFA levels in male mice from pregnancy onset until 4 months of age resulted in greater GFAP^+^ coverage in response to traumatic brain injury, an effect that was diminished when ω‐3 levels were restored (Desai et al., 2016). Treatment of cultured astrocytes with DHA was able to prevent a CORT‐induced stress response as measured by increased glutamate uptake, increased GS levels, and altered GFAP cytoskeletal morphology (Champeil‐Potokar, Hennebelle, Latour, Vancassel, & Denis, 2016). Similarly, adolescent restriction of tryptophan, an essential amino acid crucial for protein biosynthesis during development, results in astrocyte activation as shown by GFAP cytoskeletal hypertrophy in the hippocampus and amygdala immediately following tryptophan restriction (Zhang, Corona‐Morales, Vega‐González, García‐Estrada, & Escobar, 2009). While these are just some initial studies, they exemplify that restriction of essential nutrients during early‐life can impact astrocytes. Furthermore, this data shows a potential modulatory role for ω‐3 in the stress‐induced responses in astrocytes (Hennebelle, Champeil‐Potokar, Lavialle, Vancassel, & Denis, 2014).

Maternal food restriction from the last week of pregnancy until P10 resulted in enhanced astrocytic glycogen content associated with increased astrocytic GLUT‐1 in female rat cortex at P10 (Lizárraga‐Mollinedo et al., 2010). Early malnutrition provoked by early weaning resulted in elevated levels of GFAP in the hypothalamus of 6‐month‐old male rats (Younes‐Rapozo et al., 2015). However, it is unclear whether such an increase results solely from early‐malnutrition, as the prolactin injections induce central obesity, hyperglycemia, hyperleptinemia, and increased visceral fat mass in adulthood, meaning that astrogliosis could be a secondary effect of one of these features. Furthermore, injections could very well induce maternal stress, possibly resulting in altered maternal care or increased CORT levels in dams and offspring.

Together these results show that undernutrition lastingly impacts GFAP expression in astrocytes. Fatty acid restriction, tryptophan deprivation, and early weaning all resulted in increased GFAP expression (Figure 1f), while PMN has been rather shown to reduce GFAP expression (Figure 1e). This differential effect could be due to the time window of nutrient restriction. PMN was generally applied already during gestation, a period in which astrocytes have not fully developed yet, while the other models were initiated in the postnatal phase. Alternatively, the effect of undernutrition on astrocytes could be nutrient‐specific. Immediate changes induced by undernutrition included increased GS, a potential adaptive reaction to increase glutamate synthesis in response to the observed reduced glutamate uptake, and one study showed elevated GLUT1 expression and astrocytic glycogen content, suggesting energy preservation mechanisms provoked by undernutrition. Based on the described results, these metabolic alterations within astrocytes after early‐life undernutrition seem to belong to an acute adaptive response to undernutrition, rather than a persistent change in astrocyte functioning. However, since relatively little data is available on the effects of undernutrition on astrocytes, more research is necessary to draw definitive conclusions.

In general, early‐life malnutrition in the form of overnutrition or undernutrition can have a lasting impact on astrocytes. Interestingly, both overnutrition (maternal HFD, small litter size), which results in excess energy, and undernutrition (restriction of essential nutrients, early weaning), which results in lack of energy, present with a very similar phenotype, namely increased GFAP expression and possibly increased glucose transporters. It is important to consider that in the case of HFD, although energy levels remain high, a lack in (essential) nutrients might still occur. This could suggest that the observed changes are associated with alterations or shortages in circulating nutrients, changes in the metabolic profile, or just general energy disbalance, rather than it being a specific effect of either a lack or excess of energy.

### 4.4 Effects of other ELA models on astrocytes

Further supporting the sensitivity of astrocytes to ELA, there have been several reports on the effect of other forms of early stress on the astrocytic population, including dexamethasone exposure (Claessens et al., 2012; Frahm, Handa, & Tobet, 2017; McArthur, Pienaar, Siddiqi, & Gillies, 2015), early‐life exposure to noise (Jauregui‐Huerta et al., 2015; Ruvalcaba‐Delgadillo et al., 2015), restraint stress (Barros, Duhalde‐Vega, Caltana, Brusco, & Antonelli, 2006; García‐Cáceres et al., 2010), and limited nesting and bedding material during development (Gunn et al., 2013). Notably, all these models have been described to have effect on astrocytes. Due to the limited number of studies using these models, it is challenging to draw specific conclusions and speculate about the possible mechanisms involved in their effects. However, these examples highlight even further the importance of the early developmental period in determining astrocyte characteristics later in life. Clearly, at this point is key to consider the translational value and implication of the findings that we have discussed so far. However, up to date, there is very little known about how early‐life environment modulates human astrocytes. Because studying astrocyte–environment interactions during early‐life in the context of human disease is nearly impossible, there has been an emerging field of research on human‐based models to study astrocytes, like iPSC‐derived astrocytes, which enables studying astrocytes in the context of brain diseases for which ELA is a risk factor (Box 2).

## 5 CONCLUSION

In this review, we have provided initial evidence that astrocytes are acutely and permanently affected by ELA in rodents. Astrocytes undergo morphological as well as metabolic changes in response to ELA and seen their crucial role in basic brain functioning, this might affect neural functioning. Notably, the large majority of studies have focused on structural rather than functional changes in astrocytes, with a focus on GFAP expression. Then, how do we interpret changes in GFAP expression? Although upregulation of GFAP has been reported to occur in various neuropathologies (Eng, Ghirnikar, & Lee, 2000), and some functional implications of GFAP loss have been described (Liedtke et al., 1996; McCall et al., 1996; Shibuki et al., 1996; Tanaka et al., 2001), the functional consequences of alterations in GFAP expression and ELA‐induced changes in GFAP are still unclear. It is important to note that a functional defect caused by ELA can take place without a noticeable change in the GFAP astrocyte marker (Gosselin et al., 2010), indicating that ELA‐induced impairments are not always paired with changes in GFAP.

In addition, to what extent the reported alterations are implicated in ELA‐induced altered brain function needs further attention. Since astrocytes are key players in integrating a large variety of signals from the early‐life environment, and are known to play an important role in cognitive impairment and neurological dysfunction (Pekny et al., 2016; Santello et al., 2019), they might be crucial in exerting ELA‐induced effects and increasing the consequent risk of cognitive problems in humans. Moreover, human research has recently put more focus on astrocytes as a therapeutic strategy in for example MDD (see Box 1). For this reason, it should be a primary goal to unravel the underlying mechanisms that involve astrocytes in ELA‐driven risk of adult psychopathology. To this end, the emerging field and recent advances in technology (e.g., iPSC models) might be able to bring the field forward and help gain understanding of the functional relevance of these changes and their implication in ELA‐associated diseases.
